# Adenoviral Vector-Based Vaccine Platforms for Developing the Next Generation of Influenza Vaccines

**DOI:** 10.3390/vaccines8040574

**Published:** 2020-10-01

**Authors:** Ekramy E. Sayedahmed, Ahmed Elkashif, Marwa Alhashimi, Suryaprakash Sambhara, Suresh K. Mittal

**Affiliations:** 1 Department of Comparative Pathobiology, Purdue Institute for Immunology, Inflammation and Infectious Disease, Purdue University Center for Cancer Research, College of Veterinary Medicine, Purdue University, West Lafayette, IN 47907, USA; esayedah@purdue.edu (E.E.S.); aelkashi@purdue.edu (A.E.); alhashim@purdue.edu (M.A.); 2 Influenza Division, Centers for Disease Control and Prevention, Atlanta, GA 30333, USA

**Keywords:** adenoviral vector, human adenoviral vector, nonhuman adenoviral vector, influenza vaccine, universal influenza vaccine

## Abstract

Ever since the discovery of vaccines, many deadly diseases have been contained worldwide, ultimately culminating in the eradication of smallpox and polio, which represented significant medical achievements in human health. However, this does not account for the threat influenza poses on public health. The currently licensed seasonal influenza vaccines primarily confer excellent strain-specific protection. In addition to the seasonal influenza viruses, the emergence and spread of avian influenza pandemic viruses such as H5N1, H7N9, H7N7, and H9N2 to humans have highlighted the urgent need to adopt a new global preparedness for an influenza pandemic. It is vital to explore new strategies for the development of effective vaccines for pandemic and seasonal influenza viruses. The new vaccine approaches should provide durable and broad protection with the capability of large-scale vaccine production within a short time. The adenoviral (Ad) vector-based vaccine platform offers a robust egg-independent production system for manufacturing large numbers of influenza vaccines inexpensively in a short timeframe. In this review, we discuss the progress in the development of Ad vector-based influenza vaccines and their potential in designing a universal influenza vaccine.

## 1. Introduction

**Figure 1:**
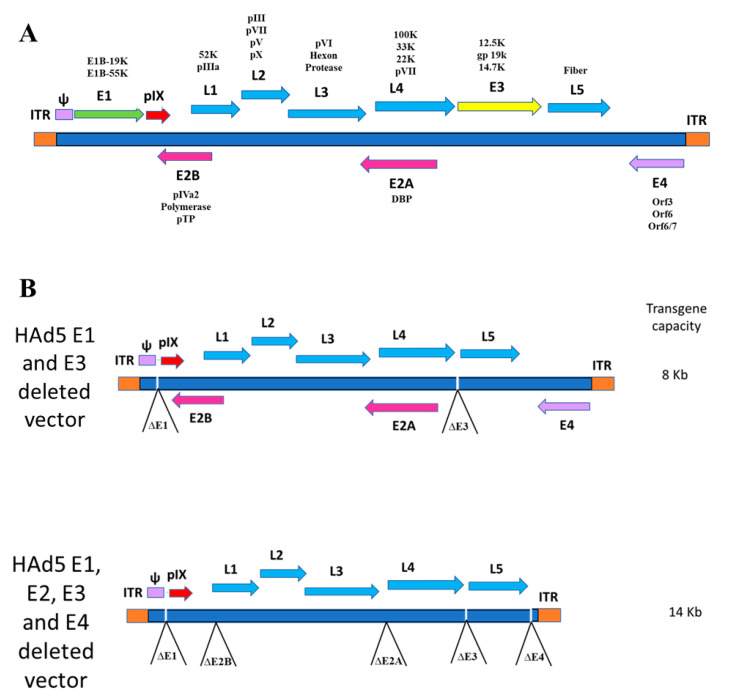
(**A**) The transcriptional map of human adenovirus type 5 (HAd5). It is composed of early (E) region (E1–E4) genes, which are responsible for genome replication, regulation of the viral transcription, and suppression of the infected cell response to the virus. The late gene transcription units (L1–L5) are expressed late in the viral replication cycle leading to the synthesis of the majority of viral structural proteins. (**B**) Diagrammatic representation of HAd5 vaccine vectors. The upper panel represents the vector genome containing the E1 and E3 deletions, and the lower panel shows the vector genome organization consisting of the E1–E4 deletions to increase the insertion capacity of foreign gene cassette.

Adenoviruses (Ads) belong to the family *Adenoviridae.* They are widely prevalent in humans, other mammals, birds, reptiles, amphibians, and fish. Out of more than 100 human Ad types, several are involved in mild respiratory infections, gastroenteritis, or conjunctivitis [1,2,3]. They are nonenveloped icosahedral viruses of approximately 90 nm in diameter with a core comprising a double-stranded linear DNA genome of roughly 25–48 kilobase pairs (kbp). The Ad genome has inverted terminal repeats (ITRs) on the right and left ends. The ITRs vary in length from 30 to 371 bp. These ITRs are essential in the initiation of the Ad genome replication. A genome packaging signal (Ψ) is found next to the left ITR, which is necessary for Ad genome packaging (Figure 1). The Ad genome encodes early genes (E1A, E1B, E2–E4) and late genes (L1–L5). The early genes are essential for the modulation of the host cell genes and the initiation of the Ad genome replication. The Ad late genes are encoding the majority of structural proteins required for the Ad capsid assembly [4].

Ad-based vectors have been used as gene delivery systems for recombinant vaccines and gene therapy applications [5]. There are many critical advantages of Ad as a gene delivery system, including the simplicity of the vector development, their ability to replicate to very high titers in cell culture, the convenience of certified cell lines for large-scale production and purification, and their safety for human applications. Moreover, they induce high levels of transgene expression, as well as high levels of antigen-specific humoral and cell-mediated immune (CMI) responses, by inducing the activation of innate immunity [6,7,8]. Ad vectors have the advantage of being delivered via either the systemic or the mucosal route. Typically, the foreign gene insertion in an Ad vector can be in any early region (E), predominately in the E1 region. To increase the transgene insertion capacity, the E1–E4 region can be deleted (Figure 1). The E3 genes are not essential for virus replication, whereas the genes in the E1, E2, and E4 regions are critical for virus replication. Therefore, for growing Ad vectors having a deletion in the E1, E2, or E4 region, there is a need for a cell line that expresses these gene cassettes to complement the viral functions for vector replication [9]. Ad vectors having only the E3 deletion are replication-competent, whereas vectors with deletion of E1, E2, E4, or any combination are replication-defective.

Influenza viruses infect humans, pigs, horses, dogs, bats, ferrets, seals, and a wide variety of birds, and belong to the family *Orthomyxoviridae* [10,11]. Human influenza viruses are grouped into A–C types. Influenza A viruses, in particular, are essential for the periodic influenza pandemics due to their prevalence in a variety of hosts, including birds. Adaptations of an avian or swine influenza A virus in humans or an antigenic shift, due to the reassortment of influenza segmented RNA genome in a mixed infection, could generate a novel influenza A virus against which humans have little to no immunity leading to an influenza pandemic. Furthermore, both influenza A and influenza B viruses undergo antigenic drifts due to immune pressure and/or the lack of proofreading ability of influenza RNA-dependent RNA polymerase. This results in the seasonal variability of influenza viruses from year to year.

## 2. Current Seasonal Influenza Vaccines and Their Potential Limitations

It is estimated that around one billion cases, including 3–5 million cases of severe illness and 290,000–650,000 influenza-related deaths occur globally every year [12]. The wide prevalence of influenza infections between humans has highlighted the significance of the disease and the immediate need for developing the next generation of influenza vaccines. On the basis of extensive influenza surveillance worldwide, influenza strains for the seasonal influenza vaccine are selected every year for better protection during the influenza season, which peaks between December and February in the USA. The majority of vaccines are still based on inactivated influenza viruses grown in embryonated eggs. For the 2020–2021 season, the egg-based trivalent vaccines contain A/Guangdong-Maonan/SWL1536/2019(H1N1)pdm09-like virus, A/Hong Kong/2671/2019(H3N2)-like virus, and B/Washington/02/2019 (Victoria lineage)-like virus, while the egg-based quadrivalent vaccines contain the same three components of the trivalent vaccine plus B/Phuket/3073/2013 (Yamagata lineage)-like virus [13]. There are two types of vaccines for the elderly in the US this season, a quadrivalent high-dose vaccine and a quadrivalent vaccine adjuvanted with MF59 [14,15].

For the 2020–2021 season, the other type of influenza vaccines is the cell- or recombinant HA-based vaccines comprising A/Hawaii/70/2019(H1N1)pdm09-like virus, A/Hong Kong/45/2019 (H3N2)-like virus, B/Washington/02/2019 (Victoria lineage)-like virus, and B/Phuket/3073/2013 (Yamagata lineage)-like virus. A quadrivalent live attenuated influenza vaccine (LAIV) as a nasal spray is also available for use in 2–49-year-old healthy individuals. 

**Figure 2:**
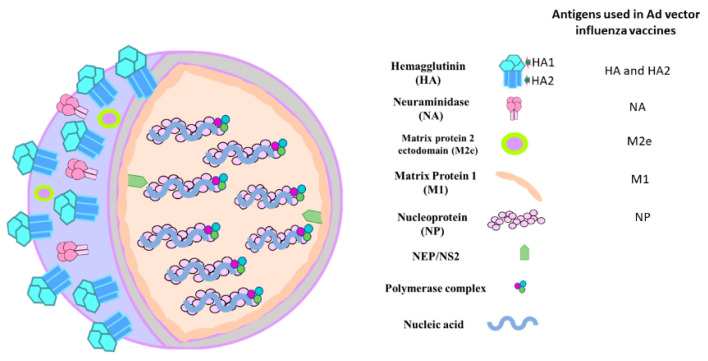
Diagrammatic sketch of influenza A virus structure. The influenza A virus is composed of eight segments of negative-sense ribonucleic acid (RNA) coupled with nucleoprotein (NP). The internal proteins include polymerase complex, nuclear export protein (NEP), and matrix 1 (M1) protein. The surface proteins are hemagglutinin (HA), neuraminidase (NA), and matrix 2 (M2) protein.

The success of the currently available seasonal influenza vaccines is mainly dependent on the match between the vaccine strains and the circulating influenza viruses during the influenza season. A significant antigenic mismatch could result in a variable degree of vaccine failure and higher incidence of influenza-like illnesses [16,17,18]. The use of GSK’s AS03 [15,19], Sanofi Pasteur’s AF03 [20], MF59 of Novartis [14,15], or Vaxine’s Advax-CpG55.2 adjuvant [21] may, on the other hand, broaden the coverage of the current influenza vaccines. The diagrammatic representation of influenza virus structural components, including the immunogenic antigens that could be used for developing Ad vector-based influenza vaccines, is shown in Figure 2.

## 3. Adjuvant Impact of Adenoviral Vectors

Unlike subunit vaccines, Ad vector-based vaccines were previously shown to elicit enhanced humoral and CMI responses [22,23], by activating innate immunity through both Toll-like receptor (TLR)-dependent and TLR-independent pathways without the need for an adjuvant [8,24]. The plasmacytoid dendritic cell (pDC)-mediated activation of innate immune response by an Ad vector is achieved through the TLR9–MyD88 pathway, which is pivotal for cluster of differentiation 8-positive (CD8^+^) T-cell activation [25]. However, non-pDC (conventional DC and macrophage)-mediated innate immune response is due to the sensing of Ad DNA in the cytoplasm. Additionally, Ad vectors induce type I interferons (IFNs), which play a crucial role in eliciting innate and adaptive immune responses [8]. Nuclear factor (NF)-κB-dependent maturation of DCs by an Ad vector is also vital for adaptive immune responses [26].

A dose-dependent secretion of proinflammatory chemokines and cytokines including interferon gamma (IFN-γ), inducible protein-10 (IP-10), macrophage inflammatory protein (MIP) alpha (MIP-α), MIP-1β, and MIP-2, C–C chemokine ligand 5 (CCL5), monocyte chemoattractant protein-2 (MCP-2), tumor necrosis factor-alpha (TNF-α), interleukin (IL)-1 beta (IL-1β), IL-6, and IL-12 were recognized following the systemic injection of Ad vectors [27,28,29,30]. These innate immune responses elicited by Ad vectors are mediated through both TLR-dependent and TLR-independent pathways [6,8,25,31]. Increased expression of TLRs 2–4, 7, and 9 with myeloid differentiation primary response 88 (MyD88) and TIR-domain-containing adapter-inducing interferon-β (TRIF) were observed following the inoculation of Ad vectors [30]. Moreover, higher expression levels of various chemokines (CCL2, CCL3, CCL4, CCL5, CXCL2, CXCL10, and IP-10) and cytokines (TNFα, IFN-γ, and IL-6) were detected early following the immunization by Ad vectors. Variable levels of innate immune responses were detected in response to human, bovine, and porcine Ad vector inoculation in mice. The bovine Ad (BAd) vector produced the highest response among those three Ad vectors [30].

In addition to the development of intended immunogen-specific immune responses, the innate immune responses also result in the induction of Ad vector-specific cellular and humoral immunity [32,33,34]. Vector-specific immune responses participate in the clearance of vector-infected cells, thereby shortening the duration of transgene expression [27,35,36,37]. Autophagy-mediated antigen presentation was discovered as a viable strategy to induce quality immune responses against vaccine antigens [38,39,40]. Various Ad vector systems may vary in their ability to undergo autophagy processes.

## 4. Significance of Ad Vector Immunity for Vaccine Platform

There are high incidences of Ad infections in humans due to the prevalence of over 100 types of Ad, leading to the development of varying levels of Ad neutralizing antibodies commonly mentioned as “Ad vector immunity” or “preexisting Ad vector immunity” [41,42]. The levels of preexisting Ad vector immunity largely depend on the exposure to Ad type(s) and vary across geographic regions and age [43,44,45]. The levels of human Ad type 5 (HAd5)-neutralizing antibody titers are in the range of 18 to 4690 [46]. HAd5-neutralizing antibody titers between 256 and 512 were observed in 16% of adults in the US [47], while a median titer of 512 in sub-Saharan children was reported [43].

The neutralizing antibodies are mainly targeted against the Ad capsid proteins (hexon, fiber, and penton), and Ad-specific CMI responses are directed against the capsid, as well as internal proteins [24,48,49]. Ad-neutralizing antibodies can neutralize the vector before internalization into the cells, whereas Ad-specific CD8^+^ T cells can eliminate the cells expressing vector proteins [50,51]. Therefore, it is anticipated that vector immunity negatively influences the duration and expression levels of the desired immunogen, as well as the quality and quantity of immune responses to the immunogen.

The impact of vector immunity on inhibiting the development of immunogen-specific immune responses in a mouse model was investigated. There was a vector immunity titer-dependent inhibition in the levels of humoral and CMI responses by the HAd5 vector expressing the hemagglutinin (HA) and nucleoprotein (NP) of A/Vietnam/1203/ 04 (H5N1) influenza virus [46]. A virus-neutralization titer of 520 resulted in a decline in the humoral and CMI responses against HA and NP but still conferred complete protection following the influenza challenge. Furthermore, a virus-neutralization titer of approximately 1500 can even be overcome either by changing the route of immunization or by increasing the vaccine dose.

In addition to the prevalence of Ad-specific immunity due to natural exposure, immunization with the Ad vector-based vaccine elicits vector-specific neutralizing antibodies in conjunction with the immunogen-specific immune responses. The acquired vector-specific neutralizing antibodies could be vital in hampering the vaccine effectiveness with the same vector if the vector neutralization antibody titers remain high. For example, seasonal influenza vaccines are required every year due to the waning of immunity against seasonal influenza viruses. Therefore, a study was conducted to investigate the effectiveness of Ad vector-based influenza vaccine annual immunization. This study examined whether Ad-neutralizing antibody titers wane to levels that do not adversely impact yearly vaccination with the same vector. The mice were mocked or HAd-primed then inoculated intramuscularly (i.m.) with 10^8^ plaque-forming units (PFU) of HAd-H5HA (HAd5 vector expressing H5N1 HA) at 1, 3, 6, and 10 months post HAd priming [52]. With time, there was a continual decline in HAd5 vector neutralization antibody titers with constant rises in the levels of HA-specific humoral and CMI responses, resulting in significant protection against challenge with an antigenically distinct H5N1 influenza virus at 6 months and onward [52]. These findings suggest that yearly immunization with the same vector is possible due to a significant drop in the vector immunity.

Due to the high seroprevalence of HAd5 in humans [24,41,44,48,53], which affects the potency of HAd5 vectored vaccines [46,52,54], rare human Ads such as HAd6, HAd11, HAd19a, HAd26, HAd28, HAd35, HAd48, and HAd49 [43,55,56,57,58,59,60,61] were developed as vectors for vaccine and gene therapy to circumvent the preexisting vector immunity. In addition, nonhuman Ad vectors (bovine Ad (BAd), chimpanzee Ad (chAd), porcine Ad (PAd), Canine Ad (CAd), and others) [47,53,62,63,64] demonstrated significant potential to overcome the preexisting vector immunity. Furthermore, the encapsulation of the Ad vector into microparticles [65,66], the coating of the vector with polyethylene glycol [66], and the alteration of Ad capsid proteins [67] were investigated to evade the preexisting vector immunity.

## 5. Ad Vector-Based Influenza Vaccines in Preclinical Trials 

### 5.1. Human Ad (HAd) Vectors in Preclinical Trials

Several HAd vectors have been used for developing influenza vaccines, but the majority of these studies were conducted with HAd5 vectors (Table 1). Many studies were performed in mice using external (HA, NA, and M2e) and internal (NP and M1) influenza virus antigens expressed in the HAd5 vector [68,69,70,71,72,73,74,75,76,77,78,79]. One of the earliest studies illustrated the partial protection of mice by the HAd5 vector carrying the HA gene [68]. In this study, mice were challenged with A/HK/1/68(H3N2) influenza virus, while the HAd5 vector expressed HA of A/Swine/Iowa/99(H3N2) virus. In another study with the HAd5 vector expressing the full HA from the A/Hong Kong/156/97(H5N1) influenza virus, the mice were completely protected against distinct strains of H5N1 influenza viruses [70] due to the development of excellent HA-specific cell-mediated and neutralizing antibody responses. On the other hand, the intranasal (i.n.) immunization of mice with HAd5 vector expressing HA of A/HK/156/97(H5N1) resulted in complete protection from the challenge with a heterologous A/VN/1203/RG(H5N1) influenza virus [80]. Similar results were reported from studies conducted on mice and ferrets [81,82,83] using HAd5 vectors. Another interesting research assessed the protection against A/Indo/05/2005(H5N1) avian influenza virus challenge in animals immunized through oral administration of HAd5 vector expressing HA of A/Indo/05/2005(H5N1) and a double-stranded RNA adjuvant [83]. The vaccinated ferrets and mice were protected from the homologous influenza virus lethal challenge. Furthermore, cross-clade neutralizing antibodies were recognized in the immunized ferrets.

The efficacies of HAd5 vector-based influenza vaccines in protecting pigs from lethal swine influenza infections were demonstrated [84,85,86]. Immunogenicity and protection efficacy of a single i.n. inoculation of HAd5-vectored vaccine expressing HA of A/CA/04/09(H1N1) was compared with the i.m. administration of the whole virus inactivated vaccine in pigs [86]. The mucosal immunoglobulin A (IgA) was induced as a result of the homologous influenza virus, in addition to cell-mediated immunity against homologous and heterologous influenza viruses in vaccinated animals. The induced immune response resulted in complete protection against the homologous influenza virus challenge and partial protection against the heterologous challenge [86].

HAd4 was used as a replication-competent vaccine vector to construct the Ad4-H5-Vtn vaccine. It contained a deletion in the E3 region to accommodate the HA gene of A/Vietnam/1194/2004(H5N1) influenza virus. The i.n. immunized mice with the Ad4-H5-Vtn vaccine survived the challenge with a lethal A/VN/PR8/CDC-RG/H5N1 virus even in the presence of preexisting immunity to the HAd4 virus [87].

### 5.2. Nonhuman Ad Vectors in Preclinical Trials

Nonhuman Ad vectors have been developed to overcome the HAd preexisting immunity in the human population [45]. Replication-defective ChAd vector-based influenza vaccines were evaluated [53,88,89,90]. The ChAd (simian AdC24 or AdC7) vector expressing the nucleoprotein (NP) of A/Puerto Rico/8/34(H1N1) influenza virus was effective in protecting BALB/c mice from two H5N1 strains [53]. A single i.n. administration of a replication-defective pan Ad type 3 (PanAd3), expressing a fusion protein of conserved NP and matrix 1 (M1) consensus sequences, was able to elicit humoral and T-cell immune responses against NP and M1 proteins [88]. The vaccinated BALB/c mice were protected from the lethal challenge of the mouse-adapted A/Fort Monmouth/1/47-ma (H1N1) virus [88]. In another study, immunization of mice with a replication-deficient AdC7 expressing HA of A/chicken/Henan/12/2004(H5N1) induced both HA-specific humoral and CMI responses and conferred complete protection against 5 LD_50_ (median lethal dose) of A/chicken/Henan/12/2004(H5N1) [89].

The replication-defective BAd3 vector-based vaccine was evaluated against highly infectious avian influenza [54,80]. BAd3 internalization is dependent on the α (2,3) or α (2,6)-linked sialic acid receptors [91], while the CAR receptors are used by most human and chimpanzee Ad vectors [92,93,94]. This feature of the BAd3 vector is attractive for mucosal immunization. A replication-defective BAd3 vector (BAd-H5HA) expressing HA of A/HK/156(H5N1) induced high levels of humoral and CMI responses in mice leading to full protection from morbidity and mortality following a lethal challenge with A/HK/483/97(H5N1) [54]. The immunogenicity and protective efficacy of the BAd-H5HA vaccine were not impacted by the exceptionally high levels of preexisting HAd5-neutralizing antibodies [54]. A low dose of BAd-H5HA vaccine (10^6^ PFU) when administered i.n. in mice conferred complete protection against an antigenically distinct A/VN/1203/RG/H5N1 influenza virus [80], whereas the i.m. immunization required a 3 × 10^7^ PFU dose of BAd-H5HA vaccine for complete protection from a heterologous influenza virus challenge. In the same study, the i.m. vaccination of mice with a 3 × 10^8^ PFU dose of HAd-H5HA failed to provide full protection following the influenza virus challenge, while i.n. immunization with a 3 × 10^7^ PFU dose of BAd-H5HA conferred complete protection. Overall, these studies demonstrate the dose-sparing superiority of the BAd3 vector compared to the HAd5 vector, even in the presence of high levels of HAd-neutralizing antibodies.

The replication-defective PAd3 vector system was also used to develop a recombinant influenza vaccine [95]. The PAd3 vector (PAV3-HA) expressing an optimized HA antigen of A/Hanoi/30408/2005(H5N1) influenza virus generated antibodies and CMI responses in mice after i.m. inoculation. The PAV3-HA vector provided better survival and lower virus load compared to the HAd5 vector with the same HA insert at 8 days and 12 months after i.m. immunization.

## 6. Ad Vector-Based Influenza Vaccines in Clinical Trials

### 6.1. Human Ad Vectors in Clinical Trials

HAd5 vector-based influenza vaccines have been used in several clinical trials [96,97,98,99,100] (Table 2). A phase II randomized influenza A challenge study (clinical trial NCT02918006) used the oral administration of an H1N1 HA HAd5 vector vaccine and a dsRNA adjuvant (VXA-A1.1) [101]. Healthy adult volunteers with undetectable or low preexisting antibodies to the H1N1 pdm09-like virus were chosen. A single dose of VXA-A1.1, a commercial injectable quadrivalent influenza vaccine (QIV), or a placebo group was used. Ninety days after immunization, vaccinated or placebo subjects were challenged with the A/H1N1 influenza virus. The outcome of this trial demonstrated 48% protection in the orally administered individuals compared to approximately 38% for those injected with QIV.

The safety and immunogenicity of an HAd5 vectored vaccine (AdhVN1203/04.H5) expressing HA of A/VN/1203/04(H5N1) were assessed in a phase I clinical trial (NCT00755703) [98]. The objective of this study was to determine the safety and immunogenicity of a two-dose i.n. vaccine at 4 weeks intervals. Three different doses (10^8^, 10^9^, and 10^10^ virus particles (VP)) were evaluated in 19–49-year-old healthy adults. The vaccine was well tolerated and induced humoral immunity as measured by hemagglutination inhibition (HI) assay [98]. Currently, a phase IIa clinical trial (NCT03232567) is ongoing with an HAd5 vector-based influenza vaccine (NasoVAX) carrying the H1HA gene as an intranasal spray [102]. The purpose of this dose-escalating study using 10^9^, 10^10^, or 10^11^ VP is to evaluate the safety and immunogenicity of the NasoVAX intranasal spray vaccine [102]. These studies signify the implication of the mucosal route of inoculation in eliciting improved mucosal protection against influenza.

The replication-competent HAd4 expressing HA of A/VN/1194/2004/H5N1 from the E3 region (Ad4-H5-Vtn) was formulated as an enteric capsule for oral administration [103,104]. A randomized, double-blind, placebo-controlled phase I clinical trial (NCT01006798) was conducted to evaluate the safety and immunogenicity of orally administered Ad4-H5-Vtn [103]. The outcomes suggested that the priming by Ad4-H5-Vtn might enhance the immunogenicity of an egg-derived H5N1 influenza vaccine.

### 6.2. Nonhuman Ad Vectors in Clinical Trials

The replication-deficient ChAdOx1 NP + M1 vaccine is based on the ChAd Y25 isolate, with deletions in E1 and E3 regions. ChAdOx1 NP + M1 is expressing NP and the matrix protein 1 (M1) of A/Panama/2007/99/H3N2 [105,106]. The safety and immunogenicity of the ChAdOx1 NP + M1 vaccine in a dose-escalation study (5 × 10^8^, 5 × 10^9^, 2.5 × 10^10^, or 5 × 10^10^ VP) were evaluated in a phase I clinical trial (NCT01818362) [107]. The significant adverse signs from mild to moderate in severity were dose-dependent and resolved within 48 h. The noticeable symptoms included pain and warmth at the site of injection, malaise, headache, and fatigue. There were dose-dependent increases in influenza virus antigen-specific T-cell responses and virus-neutralization titers. The prime-boost regimen of ChAdOx1 NP + M1 and modified vaccinia Ankara with the same insert (MVA NP + M1) lead to increased levels of humoral and CMI responses, which persisted for at least 18 months [108], suggesting the significance of prime boost with heterologous vectors for durable immunity.

## 7. Potential of Ad Vectors for Developing Universal Influenza Vaccines

The prevention and control of emerging and recurring influenza viruses require effective strategies to deal with the rapid changes in the virus antigens. Influenza viruses can evolve and evade the host immune mechanisms through two powerful phenomena: antigenic shift and antigenic drift [109,110,111,112]. Influenza pandemics have been occurring at regular intervals, and the next pandemic can happen anytime in the future. The current vaccination strategies against influenza viruses are less efficient against seasonal influenza and will not be useful in a pandemic influenza situation [16,113]. Therefore, effective novel vaccines are necessary for dealing with any potential influenza pandemic situation. The World Health Organization (WHO), the National Institutes of Health (NIH), and several other international organizations are highly supportive of developing universal influenza vaccines. These broadly protective vaccines should have the ability to confer protection against currently known and potential pandemic influenza viruses.

For developing Ad vector-based broadly protective influenza vaccines, several relatively conserved influenza proteins, including NP, M1, matrix protein 2 (M2), and the stem region of the HA protein (HA2), have been considered [75,76,114,115,116,117,118,119,120,121,122,123,124,125,126,127,128], and some are currently in clinical trials [97,99,105]. An Ad vector encoding the secreted HA2 fused with murine CD40L (rAd-SHA2FCD40L) was used for i.n. immunization of mice [117]. The vaccinated animals were protected from the lethal challenge with H1N1, H3N2, and H9N2 influenza viruses. Moreover, the sera from immunized mice were able to neutralize 13 subtypes of influenza viruses. Mice vaccinated with HAd5 vector expressing HA of A/HK/156/97 were effectively protected from the homologous challenge with A/HK/156/97, as well as from the challenge with an antigenically distinct A/VN/1203/04 virus even in the absence of effective neutralizing antibodies [70]. The inclusion of HA from two different HA subtypes in Ad vector-based vaccine formulation broadens the protective efficacy against influenza viruses [115]. The i.m. immunization of mice with HAd5 vector expressing NP of H5N1 virus resulted in approximately 2.4, 1.9, 2.3, 2.4, or 1.4 log reductions in lung virus titers of H1, H3, H5, H7, and H9 influenza viruses, respectively [115]. These results suggest the role of NP in inducing broadly protective immunity against influenza viruses. The HAd5 vector, expressing the relatively conserved domains [M2 ectodomain (M2e), HA fusion domain, HA alpha-helix domain, and a T-cell epitope of NP] of an H5N1 influenza virus, was developed and used for i.m. immunization of mice [114]. The expected humoral and/or CMI responses against these domains were observed, and 1.5, 1.2, and 1.8 log reductions in the lung viral titers of H5N2, H7N9, and H9N2 influenza virus, respectively, were observed in immunized mice following challenge [114]. These results suggest that broadly protective immune responses against influenza viruses can be induced by Ad vectors expressing selected influenza antigens or domains.

## 8. Longevity of Adenoviral Vector-Based Influenza Immunity

Generally, it is believed that immunity to influenza is only for a short duration following immunization or influenza virus infection [129]. New influenza vaccine strategies are expected to enhance the durability of protective immune responses. Humoral immune responses (HI and VN titers) in mice immunized with the HAd-H5HA vaccine regressed with time. However, significant levels were still detected even after a year, while no significant change in cellular immune responses was apparent [72]. Animals were fully protected from morbidity and mortality when challenged with a highly virulent H5N1 virus even one year after the immunization [72]. Collectively, these results suggest that Ad vector-based influenza vaccines have the potential for long-term protective immunity. Recently, we found that i.n. immunization with a BAd vector induces robust resident memory (T_RM_) and central memory T (T_CM_) cells in the lungs and spleens following vaccination (unpublished data).

## 9. Conclusions and Future Directions

The majority of currently available seasonal influenza vaccines are strain-specific. The influenza virus is prone to continuous antigen changes due to antigenic drift and occasionally antigenic shift, leading to a pandemic influenza virus. Alternatively, an avian or porcine influenza virus can undergo mutations, leading to its replication in humans and eventually efficient human-to-human transmission, thereby starting an influenza pandemic. Recently, an H1N1 swine influenza virus with pandemic potential was identified in China [130]. The yearly updated seasonal influenza vaccines produced by egg-based technology are capable of meeting the global demand under normal circumstances. In a pandemic situation, it would be almost impossible for the current influenza vaccine manufacturing technology to produce enough doses in time to meet the global demand.

The first-generation Ad vectors (with E1 and/or E3 deletions) and the second-generation Ad vectors (E1 and E3 deletions plus E2 and/or E4 deletions) are used successfully as vaccine vectors. However, the first-generation Ad vectors are more immunogenic than the second-generation Ad vectors. Ad vectors with low seroprevalence among the population are more effective and preferred as vaccine vectors. Therefore, the first-generation Ad vectors with low seroprevalence in humans (rare human Ad and non-human Ad vectors) are highly recommended for developing pre-pandemic and pandemic influenza vaccines. First-generation Ad vector-based vaccines for the Ebola virus and SARS-CoV-2 were successfully developed [131,132,133,134,135,136]. Third-generation (gutless) Ad vectors have been developed mainly for gene therapy to reduce the host immune response against the Ad vector-transduced cells. However, gutless Ad vectors have been studied as a vaccine vector for human immunodeficiency virus (HIV) [137,138].

To supplement the current influenza vaccine technology and to meet the global demand in the event of an influenza pandemic, Ad vector-based vaccine platforms have the potential to play a vital role. A large number of Ad vector-based vaccine doses at a low cost can be produced within a short timeframe. Inclusion of an immuno-stimulatory molecule with an Ad vector-based influenza vaccine can induce protective immunity one week post immunization [139] and enhance the protective efficacy for the elderly [140]. The characteristics of the next pandemic influenza virus cannot be predicted; thus, it is imperative to develop pre-pandemic vaccines and stockpile them for pandemic preparedness. The Ad vector platform has advantages over the current influenza vaccine technology to serve as a system for developing pre-pandemic vaccines, which will be critical in inhibiting the virus transmission and infection-associated morbidity and mortality during the early phase of a pandemic and before a strain-matched vaccine is available.

The availability of several human and nonhuman Ad vector systems provides versatility in Ad vector-based influenza vaccine design for developing both seasonal and pandemic vaccines. In addition to the humoral immune response, the importance of the CMI response in generating broader protection has been realized. The role of NP-specific immune responses in protection has been demonstrated. Along with NP, the relatively conserved protective epitopes have been identified in HA2 and M2e domains; therefore, the probability of designing a universal influenza vaccine seems very high. Additional work is needed to explore whether Ad vector-based influenza vaccines could be used in all segments of the population. The use of the prime-boost approach with two different Ad vectors [54,141], Ad and another viral vector [142,143,144,145,146,147], or Ad vector and DNA immunization [148,149,150,151] will confer a considerably higher level of immune responses, especially in the elderly.

## Figures and Tables

**Table 1:** Preclinical trials with adenoviral vector-based influenza vaccines.

Vector	Gene Insertion Site	Immunogen	Dose	Route of Inoculation	Immune RESPONSEEVALUATED	Host	Challenge Virus	Protection Level	References
HAd5	E1	HA [A/Swine/Iowa/1999(H3N2)]	5× 10^8^ TCID50	IM	Humoral	Mice	[A/HK/1/1968(H3N2)]	Partial	[69]
HAd5	E1	HA [A/Hong Kong/156/1997(H5N1)]	1 × 10^8^ PFU	IM	Humoral, CMI	Mice	A/HK/483/1997 (H5N1) A/VN/1203/200 (H5N1) A/HK/213/2003 (H5N1)	Complete	[71]
HAd5	E1	HA [VN/1203/2004(H5N1)] HA [HK/156/1997(H5N1)]	5 × 10^10^ VP	IM, SC, IN	Humoral, CMI	Mice, Chicken	A/VN/1203/2004(H5N1)	Complete	[70]
HAd5	E1	HA [A/Ca/4/2009 (H1N1)]	5 × 10^10^ VP	IM	Humoral, CMI	Mice	A/Ohio/7/2009(H1N1)	Complete	[72]
HAd5	E1	HA [A/HK/156/1997 (H5N1)]	Multiple	IM	Humoral, CMI	Mice	A/HK/483/1997(H5N1)	Complete	[73]
HAd5	E1	HA and NP [VN/1203/2004 (H5N1)]	1 × 10^8^ PFU	IM	Humoral, CMI	Mice	PR8 reassortant A/Indo/05/2005(H5N1) A/VN/1203/2004(H5N1)	Complete	[74]
HAd5	E1	NP [A/PR/8/1934(H1N1)] NP [B/Ann Arbor/1/1986)	1 × 10^10^ VP	IM	Humoral, CMI	Mice	A/PR/8/1934(H1N1) A/HK/483/1997(H5N1) A/HK/156/1997(H5N1)	Partial	[76]
HAd5	E1	M2 consensus sequence	1 × 10^10^ VP	IM	Humoral, CMI	Mice	A/PR/8/1934(H1N1) A/Thailand/SP-83/2004 (H5N1)	Complete	[77]
HAd5	E1	M2 consensus sequence NP [A/PR/8/193 (H1N1)] NP [B/Ann Arbor/1/1986)	1 × 10^10^ VP	IM	CMI	Mice	A/PR/8/1934(H1N1)]	Complete	[150]
							A/VN/1203/2004(H5N1)	Partial	
HAd5	E1, E3, E4	HA, NP, and M2 A/Thailand/1/KAN-1/2004	1 × 10^10^ VP	IM	Humoral	Mice Ferrets	A/VN/1203/2004(H5N1)	Complete	[82]
HAd5	E1	HA and NP A/Swine/Iowa/1999(H3N2)	2× 10^10^ TCID50	IM	Humoral	Pig	A/Swine/Iowa/1999(H3N2)	Complete	[85]
AdC7	E1	NP[A/PR/8/1934 (H1N1)]	1 × 10^11^ VP	IM	CMI	Mice	A/VM/1203/2004(H5N1) A/HK/483/1997(H5N1)	Partial	[53]
BAd3	E1	HA [A/HK/156/1997 (H5N1)]	1 × 10^8^ PFU	IM	Humoral, CMI	Mice	[A/Hong Kong/483/1997(H5N1)]	Complete	[54]
BAd3	E1	HA [A/HK/156/1997 (H5N1)]	Multiple	IM, IN	Humoral, CMI	Mice	A/Vietnam/1203/2004(H5N1)-PR8/CDC-RG	Complete	[81]

HAd5, human adenovirus type 5; AdC7, chimpanzee adenovirus type 7; BAd3, bovine adenovirus type 3; E1, early region 1; E3, early region 3; HA, hemagglutinin; NP, nucleoprotein, M2, matrix 2; VP, virus particles; TCID50, tissue culture infectious dose 50; PFU, plaque-forming units; IM, intramuscular; IN, intranasal; CMI, cell-mediated immunity.

**Table 2:** Adenovirus vector-based influenza vaccines in clinical trials.

Vector	Vector Deletions	Insert	Phase	Route	Clinical Trial Number	Sponsor
Human adenovirus type 4 (HAd4)	Partial E3 deletion	H5HA	I	Oral	NCT01006798	PaxVax
			I	Intranasal	NCT01806909	PaxVax
			I	Oral, tonsillar	NCT01443936	PaxVax
Human adenovirus type 5 (HAd5)	E1 and E3	H1HA+ dsRNA	II	Oral + H1N1 challenge	NCT02918006	VaxArt
			I	Oral	NCT01688297	VaxArt
			I	Oral	NCT03121339	VaxArt
			I	Ileum radio-controlled capsule	NCT01761123	VaxArt
		H5HA+ dsRNA	I	Oral	NCT01335347	VaxArt
		H1HA	IIa	Intranasal	NCT03232567	Altimmune
		H5HA	I	Intranasal	NCT00755703	Vaxin/Altimmune
Chimpanzee adenovirus (ChAd) + Modified Vaccinia Ankara (MVA)	E1 and E3	NP+M1 of H3N2	I	IM heterologous prime-boost	NCT01623518	Jenner Institute
			I	IM heterologous prime-boost	NCT01818362	Jenner Institute

E1, early region 1; E3, early region 3; H5HA, hemagglutinin of H5N1 virus; H1HA, hemagglutinin of H1N1 virus; NP, nucleoprotein; M1, matrix 1; IM, intramuscular.
